# Relating CYP2B6 genotype and efavirenz resistance among post-partum women living with HIV with high viremia in Uganda: a nested cross-sectional study

**DOI:** 10.1186/s12981-023-00514-2

**Published:** 2023-03-31

**Authors:** Allan Buzibye, Kara Wools-Kaloustian, Adeniyi Olagunju, Ellon Twinomuhwezi, Constantin Yiannoutsos, Andrew Owen, Megan Neary, Joshua Matovu, Grace Banturaki, Barbara Castelnuovo, Mohammed Lamorde, Saye Khoo, Catriona Waitt, Agnes Kiragga

**Affiliations:** 1 Research Department, Infectious Diseases Institute, College of Health Sciences, Makerere University, P. O. Box 22418, Kampala, Uganda; 2 School of Medicine, Infectious Diseases, Indiana University, Indianapolis, IN USA; 3 Department of Pharmacology and Therapeutics, University of Liverpool, Liverpool, UK; 4 Fairbanks School of Public Health, Indiana University, Indianapolis, IN USA

**Keywords:** HIV, Pregnancy, Post-partum, *CYP2B6*, HIV drug resistance, Single nucleotide polymorphisms

## Abstract

**Background:**

We investigated the association between *CYP2B6* polymorphisms and efavirenz drug resistance among women living with HIV who started on antiretroviral therapy during pregnancy and with high viremia during post-partum.

**Methods:**

This was a cross-sectional study of women with viral loads greater than 1000 copies/ml who were at least 6 weeks postpartum. Sanger sequencing was used to detect resistant mutations, as well as host genotyping, and efavirenz resistance was compared among the metabolizer genotypes.

**Results:**

Over the course of one year (July 2017-July 2018), 322 women were screened, with 110 (34.2%) having viral loads of 1000 copies/ml and 62 having whole blood available for genotyping. Fifty-nine of these women had both viral resistance and human host genotypic results. Efavirenz resistance according to metabolizer genotype was; 47% in slow, 34% in extensive and 28% in intermediate metabolizers, but the difference was not statistically significant due to the small sample size.

**Conclusions:**

There was no statistically significant difference in EFV resistance between EFV metabolizer genotypes in women who started antiretroviral therapy during pregnancy and had high viremia in the postpartum period. However, a numerical trend was discovered, which calls for confirmation in a large, well-designed, statistically powered study.

## Background

Improved access to antiretroviral therapy (ART) by pregnant and post-partum women has significantly contributed to prevention of transmission of HIV infection to children, with 52% global reduction between 2010 and 2019 [1]. However, attrition from care and treatment suboptimal adherence during pregnancy and breastfeeding is still associated with mother to child transmission (MTC). In 2012, World Health Organisation (WHO) recommended starting efavirenz (EFV)-based regimens for life in pregnant women for the Prevention of MTC (PMTCT) transmission [2]. With advances in HIV therapy, dolutegravir (DTG) replaced EFV due to its efficacy and safety profiles in 2019 [3]. However, EFV remains an option for patients in whom DTG is contraindicated. A major clinical challenge with EFV is neuropsychiatric events, which may contribute to poor adherence in some individuals [4].

Efavirenz is metabolized to 8-hydroxy-efavirenz by cytochrome P450 (CYP) monooxygenase system, predominantly by CYP2B6 [5]. Single nucleotide polymorphisms (SNPs) in CYP2B6 gene lead to different drug metabolizer phenotypes, with slow and fast metabolizer phenotypes being disadvantageous and contributing to secondary resistance. Slow metabolizers may experience toxic drug concentrations which could contribute to maladherence [6, 7] while extensive metabolizers are at risk of sub-therapeutic concentrations [8].

Among CYP2B6 polymorphisms, 516 and 983 causing slow metabolism are more frequent among individuals of African ancestry as compared to Caucasians [9, 10]. However, no study has reported the association between CYP2B6 polymorphisms and EFV resistance in HIV post-partum women living with high viremia. We hypothesized that interrupting EFV therapy due reasons like treatment suboptimal adherence increases the risk of HIV resistance mutations.

## Methods

### Study design

This was a cross-sectional study nested in “Tracing non-rEtained HIV Positive pregnant Women enrolled in option B + and ascertaining their babIeS outcomes (sTEPWISe)” study conducted between July 2017 and July 2018 [11].

### Study site and population

The sTEPWISe study was conducted at HIV clinics of Kampala City Council Authority which are supported by the Infectious Diseases Institute (IDI) through CDC-PEPFAR. Study activities leveraged on routine services offered at the PMTCT clinics for newly diagnosed pregnant Women Living With HIV (WLHIV). The study aimed to trace pregnant WLHIV initiated on ART during pregnancy and disengaged from care, and were at least 6–12 weeks postpartum. Adherence to ART was self-reported and determined by clinicians as good, fair, or poor if participant reported missing < 2, 2–4, and ≥ 5 doses per month respectively. A participant was defined as disengaged from care if no clinic visit was recorded 90 days preceding database closure.

### Sampling method

A random sample of retained mothers with viralogical failure (viral load ≥ 1000 copies/ml) was tested for presence of SNPs that influence plasma EFV concentration exposure and HIV resistance mutations to EFV. Seven participants were also randomly selected for EFV drug level measurement to confirm adherence status. A random sample of women disengaged from care were contacted by telephone and home visited to consent for blood draw for the aforementioned tests.

### Laboratory studies

Drug resistance testing was performed at the Infectious Diseases Institute Core Laboratory, Kampala, Uganda, which is accredited by the College of American Pathologists (CAP). Viral RNA was isolated from plasma samples using the Invitrogen Purelink viral RNA/DNA kit (Thermo Fisher Scientific, Dreieich, Germany). HIV-1 genotyping was performed on extracted RNA using an in-house HIV-1 genotyping assay covering the protease (PR) and reverse transcriptase (RT) with subsequent sequencing using the BigDye Terminator v3.1 Cycle Sequencing Kit (Thermo Fisher Scientific, Dreieich, Germany). Sequence analysis was performed using AB3730 capillary sequencer (Thermo Fisher Scientific, Dreieich, Germany).

Genotyping of the three CYP2B6 polymorphisms that predict increased EFV exposure was undertaken at the University of Liverpool. The E.Z.N.A. Blood DNA Mini Kit (Omega Bio-Tek Inc., Norcross, Georgia, USA) was used to extract genomic DNA, which was then quantified using NanoDrop (Thermo Fisher Scientific Inc., Wilmington, Delaware, USA) and stored at − 20 °C until analysis. A real-time PCR allelic discrimination assay on a DNA Engine Chromo4 system was used for genotyping (Bio-Rad Laboratories Inc., Hercules, California, USA). The PCR protocol involved denaturation of DNA at 95 °C for 10 min, 40 cycles of amplification at 95 °C for 15 s and annealing at 60◦C for 1 min. TaqMan Genotyping Master Mix and assays for CYP2B6 (516G → T [*rs3745274*], 983 T → C [*rs28399499*], and 15582C → T [*rs4803419*]) were obtained from Life Technologies Ltd (Paisley, Renfrewshire, UK). Allelic discrimination plots and genotype assignments were performed using Opticon Monitor, version 3.1 (Bio-Rad Laboratories Inc.).

### Analysis

Drug resistance-associated mutations in protease and reverse transcriptase gene regions were interpreted using the Stanford HIV database (HIVdb) program. To predict susceptibility to EFV a resistance score was calculated and classified as susceptible, low, intermediate, and high-level following the Stanford HIVdb scoring system (http://hivdb.stanford.edu) [13] Metabolizer genotype was categorized according to the ACTG algorithm as slow, intermediate, and extensive. We used multivariate logistic model to assess association between EFV resistance and metabolizer genotypes.

## Results

### Participants

Three hundred and twenty two women were enrolled between July 2017 and July 2018 into the parent sTEPWISe study of whom 110 (34.2%) had viral load ≥ 1000 copies/ml. For this sub-study, 62 of 110 women provided whole blood samples for genotyping. Of 62 samples, 3 failed to amplify, 10 were from women retained in care and 49 disengaged from care.

At ART initiation, median age was 25 years (interquartile range [IQR]: 22-28), median weight was 60 kg (IQR: 50,− 80), median CD4 T-cell count was 516 cells/mm3 (IQR: 324-640), median viral load was 25,562 copies/ml (IQR: 6,117-59,480) and median duration between ART initiation and viral load testing was 10 months (IQR: 8-11).

Over 90% (55/59) of participants were in their second or third trimester. Good adherence was reported at 90% by the retained women and 51% of the disengaged. Of the seven participants selected for EFV drug level measurement, none had detectable drug levels (disengaged-6, retained-1). All the 59 participants received EFV, tenofovir disproxil fumerate and lamivudine throughout the study period except one participant that received emtricitabine instead of lamivudine. The CYP2B6 population genotypic frequencies were; 30%, 50%, and 20% for slow, intermediate, and extensive phenotypes, respectively.

### HIV Viral resistance results

**Fig.1:**
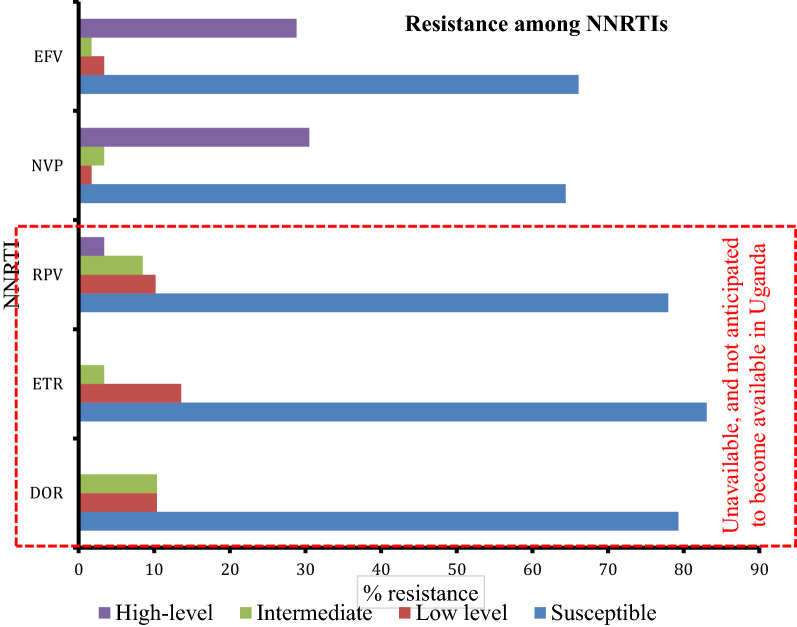
Non-nucleoside reverse transcriptase inhibitors resistance among post-partum women living with HIV with high viremia. *EFV* efavirenz, *NVP* nevirapine

Figure 1 shows resistance to non-nucleoside reverse transcriptase (NNRTIs) inhibitors stratified by phenotype. The overall prevalence of EFV resistance was 34% (20/59).

K103N conferring resistance to EFV and NVP was more common, occurring in 11/20 participants with resistance followed by E138A detected in two participants. M184V was the only clinically relevant mutation that renders resistance to NRTIs detected in the study.

### Comparison of efavirenz resistance and host genotype

Table 1 shows the frequency of EFV resistance stratified by genotype. There was no statistically significant difference in the efavirenz resistance between metabolizer genotypes and between retained and disengaged women.

**Table 1:** Comparison of efavirenz resistance stratified by different host genotypes

Metabolizer category	n	EFV resistance n (%)	Crude OR (95% CI)	P-value	Adjusted OR (95% CI)	P-value
Slow	17	8 (47.1)	2.3(0.7–8.2)	0.2	2.3(0.6–8.2)	0.2
Intermediate	29	8 (27.6)	1.0		1.0	
Extensive	13	4 (30.8)	1.2(0.3–4.9)	0.8	1.1(0.3–4.6)	0.9
Mother status						
DW	49	15(36.6)	1.0			
RW	10	5(50.0)	2.3(0.6–9.0)	0.2	2.3(0.6–9.4)	0.3

The results of three participants are missing because they failed to amplify. DW-disengaged women, RW-retained women

*EFV* efavirenz, *OR* odds ratio, *CI* confidence interval

## Discussion

The prevalence of virological failure among Ugandan post-partum women initiated on ART during pregnancy was 34.2%. Overall EFV resistance was 34% with slow metabolizers (47%) having a higher frequency than extensive (31%) and intermediate (28%) women.

Virological failure rates observed in this study were higher than observed in other studies in Africa [14–16]. However, unlike most published studies, this study in addition to retained women sought to find women who had been lost to follow-up. According to a South African study [17], 8.4% of lost-to-follow-up post-partum women were not on treatment, while 25.1% re-engaged following treatment interruption. Treatment interruption leads to high rates of resistance, rendering virologic suppression nearly impossible with re-initiation of first-line regimens when re-engaged in care (for example, during a future pregnancy) [18]. If not addressed, this may increase perinatal transmission and hamper the efforts of prevention of mother-to-child transmission.

Prevalence of resistance to EFV reported in this study is similar to that reported from Malawi in women initiated on ART while pregnant, with 35% prevalence among patients with viral load ≥ 1000 copies/ml. However, a study undertaken in Tanzania reported higher rates of NNRTI resistance (57%) among pregnant women who started on nevirapine [17]. The authors ascribed the elevated resistance rates in the Tanzanian research to poor adherence. Self-perceived adherence was, however, higher than reported adherence in our study even at 24 months postpartum. It's possible that the women didn’t report accurately. Inaccurate self-reported adherence was confirmed by undetectable plasma EFV concentrations measured in this study.

There was no statistically significant difference in EFV resistance among metabolizer genotypes. This contradicts the findings of a Botswana study, which found a link between EFV/NVP resistance and the CYP2B6 516G allele (OR: 2.26; 95% CI 1.27–4.01; P = 0.005) [18]. The aforementioned study was a case–control study of HIV patients on either an EFV or NVP-based regimen who were not post-partum women. The justification for the study objectives, however, is similar to ours. The Botswana study, unlike ours, was statistically powered to detect a statistical difference.

### Limitations and strengths of the study

The study sample size was small to draw a statistically significant conclusion. This study did not assess whether viral resistance was primary or secondary, yet high frequencies of primary resistance could have greatly influenced study conclusions.

## Conclusions

We did not observe a statistically significant difference in EFV resistance between EFV metabolizer genotypes. However, a numeric trend was observed that warrantees confirmation in a large well designed statistically powered study.

## Data Availability

The data that support the findings of this study are available from the sTEPWISe study team but restrictions apply to the availability of this data and are so not publicly available. Data is however available from the corresponding author upon reasonable request and with permission of the sTEPWISe study team.

## Abbreviations

EFV: Efavirenz
ART: Antiretroviral therapy
AIDS: Acquired immunodeficiency syndrome
WHO: World Health Organisation
HIV: Human immunodeficiency virus
UNAIDS: Joint United Nations Programme on HIV and AIDS
DTG: Dolutegravir
ACTG: AIDS clinical trials group
NRTI: Nucleotide reverse transcriptase inhibitor
CNS: Central nervous system
RNA: Ribonucleic acid
DNA: Deoxyribonucleic acid
IDI: Infectious diseases institute
WLHIV: Women living with HIV
VL: Viral load
CAP: College of American Pathologists
PCR: Polymerase chain reaction
USA: United States of America
NNRTIs: Non-nucleotide reverse transcriptase inhibitors
PIs: Protease inhibitors
TDF: Tenofovir
3TC: Lamivudine
OR: Odds ratio
CI: Confidence interval
IQR: Interquartile range
